# Giant Fibroid Mimicking a Gastrointestinal Stromal Tumor: A Diagnostic Dilemma

**DOI:** 10.7759/cureus.35883

**Published:** 2023-03-07

**Authors:** Gayathri Priyadharshinee, Tapas K Sahu, Channabasappa Chavadi

**Affiliations:** 1 Radiology, Manipal Hospitals, Bangalore, IND; 2 Radiology, Kalinga Institute of Medical Sciences, Bhubaneswar, IND

**Keywords:** abdominopelvic mass, uterine mass, intra-abdominal mass, abdomino-pelvic ct, transabdominal ultrasound, radiological dilemma, extraintestinal gist, gastrointestinal stromal tumor (gist), uterine fibroid

## Abstract

Uterine fibroids are the most common benign pelvic tumors in females of the reproductive age group. Usually, fibroids are confined to the uterus. Here, we report an interesting and rare case of a large 17 cm abdominopelvic mass lesion that led to a diagnostic dilemma between a mesenteric gastrointestinal stromal tumor (GIST) and a uterine fibroid. We had a 26-year-old female who underwent an ultrasound examination of the abdomen as the initial imaging modality and was found to have an abnormally large solid abdominopelvic mass lesion. For further evaluation, a contrast-enhanced CT examination of the abdomen-pelvis was done. Initially, on first look, the mass was thought to be of mesenteric origin, but on further review of images, it was found to be of gynecological origin. Intraoperatively, the solid mass was seen attached to the uterine fundus and underwent excision. Histopathological examination confirmed the mass to be a uterine fibroid. This case study describes the uncommon appearance of this tumor in a young woman, including the clinical presentation, imaging, and surgical findings.

## Introduction

Uterine fibroids are benign tumors that arise from the smooth muscles and fibroblasts of the myometrium. They are a common gynecological condition affecting women of reproductive age. On the other hand, gastrointestinal stromal tumors (GISTs) are rare mesenchymal tumors that arise from the interstitial cells of Cajal in the gastrointestinal tract. Also, there are extra-GISTs that are known to occur in the mesentery, omentum, and retroperitoneum. Although both fibroids and GISTs are benign, they can present with similar symptoms, including abdominal pain, bloating, and an abdominal mass.

Diagnostic challenges can arise when fibroids and GISTs exhibit similar clinical and radiographic features, leading to misdiagnosis or delayed diagnosis. This case report presents a unique case of a fibroid mimicking a GIST, highlighting the importance of thorough clinical evaluation and imaging studies for accurate diagnosis and management. This report aims to increase awareness of this uncommon entity and provide insights into the diagnostic and therapeutic approach to similar cases.

## Case presentation

A 26-year-old female presented to the Department of Internal Medicine with vague complaints of abdominal bloating and nonspecific abdominal pain for the last two months. The patient had no history of significant weight loss or gain. Gynecological history revealed her to be a nulliparous female with regular cycles and no complaints of excess bleeding or pain. On examination, a large firm mass was palpable in the abdomen and pelvis. Per vaginal examination was unremarkable with free fornices and no tenderness.

Imaging findings

**Figure 1:**
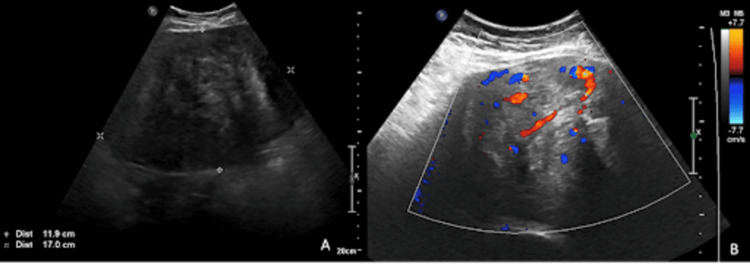
B-mode transabdominal ultrasound image (A) and color Doppler image (B) demonstrating a large well-demarcated heterogeneously hypoechoic abdominopelvic mass lesion with internal vascularity.

**Figure 2:**
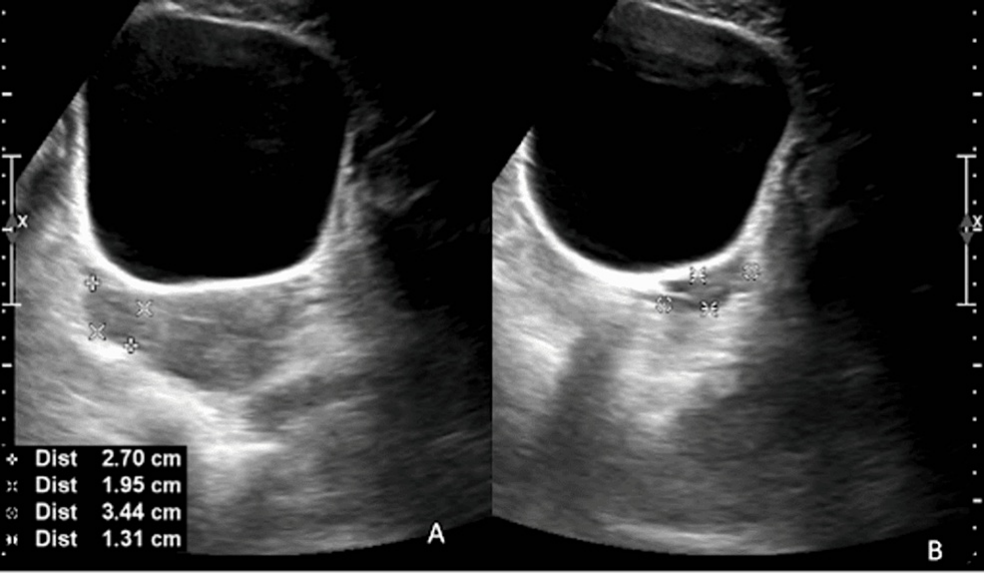
B-mode transabdominal ultrasound images reveal that the right (A) and left (B) ovaries are visualized separately.

An ultrasound examination of the abdomen was done as the initial imaging modality, which revealed a large solid abdominopelvic mass lesion of size 17 x 11.9 cm (Figure 1). The mass was separate from the liver and the pancreas. The ovaries could be separately visualized in the transabdominal study (Figure 2). Unfortunately, the patient did not consent to transvaginal examination.

**Figure 3:**
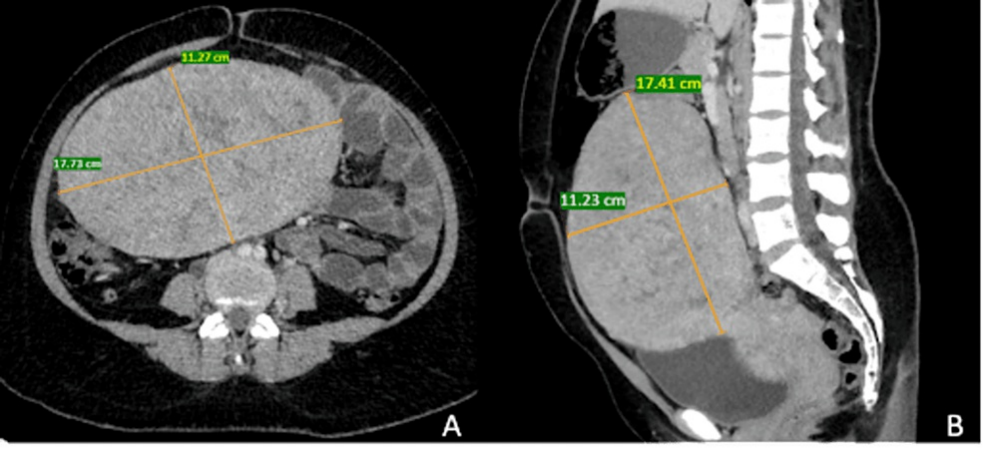
Axial (A) and sagittal (B) sections of contrast-enhanced CT. A large well-defined abdominopelvic solid mass lesion is seen with heterogeneous enhancement. The mass effect in the form of displacement of adjacent bowel loops, mesenteric fat, and omental fat is noted. Craniocaudally, the mass extends from the subgastric region to the uterine fundus.

**Figure 4:**
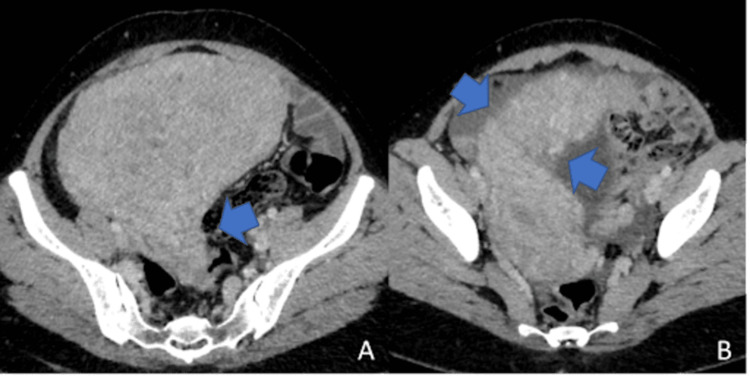
Axial sections of contrast-enhanced CT abdomen-pelvis at different levels (A and B) reveal the “pedicle sign” (blue arrows) connecting the inferior end of the large mass to the right adnexa.

To further characterize the mass, a contrast-enhanced CT examination of the abdomen and pelvis was done. The abdominopelvic solid mass lesion having its epicenter in the mid-abdomen (Figure 3), rather than in the pelvis, favored a diagnosis of mesenteric GIST. Upon a closer look at the images, the mass demonstrated the pedicle sign, which favored gynecological origin (Figure 4). There was no significant adenopathy or free fluid.

Treatment and outcome

**Figure 5:**
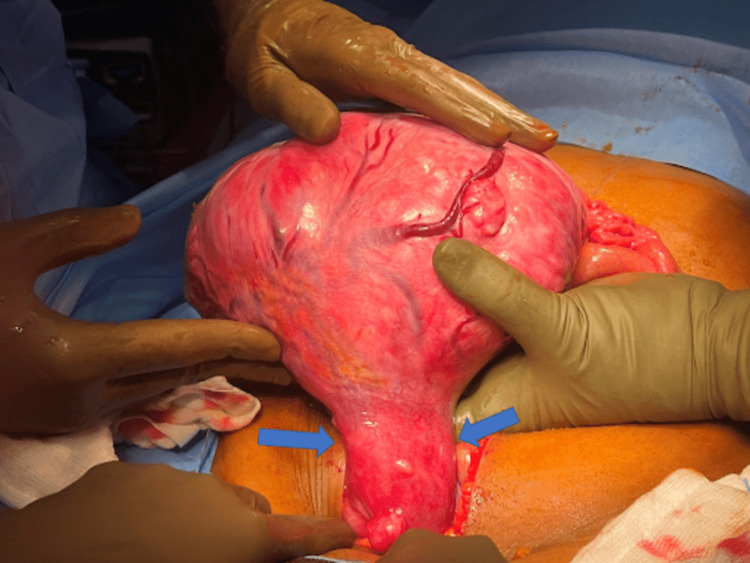
Intraoperative image demonstrating the "pedicle sign" (blue arrows): the large abdominopelvic mass is attached to the uterine fundus, consistent with a subserous pedunculated fibroid.

The routine blood reports were normal, including tumor markers. She underwent laparotomy, and intraoperatively, the solid mass was seen attached to the uterine fundus in subserosal location with a pedicle (Figure 5). The mass was mobilized without complications, and the pedicle was excised. The myometrial tissue was spared, owing to its pedunculated subserosal location. Histopathological examination confirmed the mass to be a uterine fibroid. She was subsequently discharged and is currently doing well.

## Discussion

In large abdominopelvic masses, it can be difficult to determine the organ of origin. The first step is to determine if the mass is intraperitoneal or extraperitoneal [1].

Literature has described signs that help localize the epicenter: The presence of a pedicle connecting the mass to the organ of origin is “pedicle sign,” as seen in this case [2]. A few other signs are the "beak sign," a sharp angle between the ovary and the mass forms a beak shape at the edge of the ovary; the "phantom organ sign," nonvisualization of an organ in the context of no appropriate surgical history; the embedded organ sign, the organ of origin appears embedded in the mass. 

A review of the literature has shown that a diagnostic dilemma prevails in cases similar to this. There are reported incidences where suspected gynecological neoplasms turned out to be GISTs [3-6]. Morimura et al. [3] reported two cases of GISTs mimicking gynecologic neoplasms (preoperative diagnoses of ovarian fibrothecoma and subserosal leiomyoma). Similarly, Lee [4] reported a proven case of GIST that was initially diagnosed as ovarian cancer. Other differentials include cystic degeneration of fibroid [5], leiomyosarcoma, liposarcoma, desmoid, schwannoma, etc. [6]. See et al. [7] reported a similar case where a possible GIST was intraoperatively found to be a fibroid. Also, it can be challenging to differentiate an adnexal fibroid from an ovarian mass [8,9]. 

In routine practice, uterine fibroids are easily diagnosed with ultrasound. The presentation ranges from asymptomatic to malignant mimics [4,10]. Fibroids are benign neoplasms that do not require aggressive treatment. On the other hand, GISTs are uncommon tumors of the gastrointestinal system and may require chemotherapy or radiation in addition to surgery. 

Both fibroids and GISTs can have heterogeneous appearances on imaging: Fibroids can undergo degenerative changes and GISTs can undergo malignant transformation or necrosis when large. Currently, there are no recommendations regarding the best imaging modality to diagnose GISTs. The diagnosis is made intraoperatively or with histological examination. 

## Conclusions

This case report is to emphasize the need for radiologists and clinicians to be aware of the possibility of GIST mimicking a fibroid or vice versa in order to avoid misdiagnosis and to provide appropriate care for the patient. The reporting radiologist should keep gynecologic neoplasms in their list of differentials in cases where the symptoms and imaging results of a GIST are atypical. The accurate diagnosis of the mass lesion (GIST or fibroid) is essential for proper treatment planning, prognostication, treatment, and potentially negative outcomes for the patient. Further investigation, including biopsy, may be necessary to differentiate between the two conditions in certain cases.
